# Role of TRPV1 in electroacupuncture‐mediated signal to the primary sensory cortex during regulation of the swallowing function

**DOI:** 10.1111/cns.14457

**Published:** 2023-09-18

**Authors:** Si Yuan, Bo Qiu, Ying Liang, Bing Deng, Jing Xu, Xiaorong Tang, Junshang Wu, Sheng Zhou, Zeli Li, Hongzhu Li, Qiuping Ye, Lin Wang, Shuai Cui, Chunzhi Tang, Wei Yi, Lulu Yao, Nenggui Xu

**Affiliations:** ^1^ South China Research Center for Acupuncture and Moxibustion, Medical College of Acu‐Moxi and Rehabilitation Guangzhou University of Chinese Medicine Guangzhou China; ^2^ Department of Rehabilitation of Traditional Chinese Medicine Hunan University of Chinese Medicine Changsha China; ^3^ Rehabilitation Center First Affiliated Hospital of Guangzhou University of Chinese Medicine Guangzhou China; ^4^ Department of Rehabilitation Medicine, The Third Affiliated Hospital Sun Yat‐sen University Guangzhou China; ^5^ Research Institute of Acupuncture and Meridian, College of Acupuncture and Moxibustion Anhui University of Chinese Medicine Hefei China

**Keywords:** electroacupuncture, post‐stroke dysphagia, swallowing function, TRPV1

## Abstract

**Aims:**

Electroacupuncture (EA) at the Lianquan (CV23) could alleviate swallowing dysfunction. However, current knowledge of its neural modulation focused on the brain, with little evidence from the periphery. Transient receptor potential channel vanilloid subfamily 1 (TRPV1) is an ion channel predominantly expressed in sensory neurons, and acupuncture can trigger calcium ion (Ca^2+^) wave propagation through active TRPV1 to deliver signals. The present study aimed to investigate whether TRPV1 mediated the signal of EA to the primary sensory cortex (S1) during regulation of swallowing function.

**Methods:**

Blood perfusion was evaluated by laser speckle contrast imaging (LSCI), and neuronal activity was evaluated by fiber calcium recording and c‐Fos staining. The expression of TRPV1 was detected by RNA‐seq analysis, immunofluorescence, and ELISA. In addition, the swallowing function was assessed by in vivo EMG recording and water consumption test.

**Results:**

EA treatment potentiated blood perfusion and neuronal activity in the S1, and this potentiation was absent after injecting lidocaine near CV23. TRPV1 near CV23 was upregulated by EA‐CV23. The blood perfusion at CV23 was decreased in the TRPV1 hypofunction mice, while the blood perfusion and the neuronal activity of the S1 showed no obvious change. These findings were also present in post‐stroke dysphagia (PSD) mice.

**Conclusion:**

The TRPV1 at CV23 after EA treatment might play a key role in mediating local blood perfusion but was not involved in transferring EA signals to the central nervous system (CNS). These findings collectively suggested that TRPV1 may be one of the important regulators involved in the mechanism of EA treatment for improving swallowing function in PSD.

## Graphical abstract

The upregulation of TRPV1 at the CV23 acupoint mediates local blood perfusion rather than transmission of EA signals to the central nervous system, which potentially contributes to improving swallowing function in PSD following EA treatment.

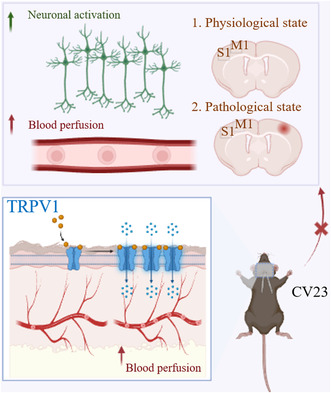

## 1 INTRODUCTION

Stroke is the most common disease that disrupts the swallowing network, resulting in post‐stroke dysphagia (PSD).^1^, ^2^, ^3^, ^4^, ^5^ Electroacupuncture (EA) is a traditional acupuncture with contemporary electrotherapy technologies and utilized as an effective intervention for PSD.^6^, ^7^, ^8^ However, the mechanism of the EA's effect is still in the exploration. Previous investigations have reported that neural activity in the primary motor cortex (M1) was enhanced by EA‐CV23 in PSD mice.^8^, ^9^, ^10^, ^11^ When acupuncture is delivered to acupoints, the cortex is activated.^12^, ^13^ Nevertheless, the pathway of EA signals transmitting to M1 remains unclear. The primary sensory cortex (S1) plays a significant role in sensory input processing.^14^ Unilateral infarction of the M1 results in impaired blood flow and neuronal interactions between the contralateral M1 and S1, which is critical for the recovery during EA at Baihui and Dazhui treatment.^15^ Therefore, we hypothesized that S1 may be involved in transmitting EA signals to M1 and regulating swallowing function.

Acupoints, situated in deep tissue with numerous sensory nerve terminals, exhibit a strong correlation with peripheral sensory afferents.^16^, ^17^, ^18^, ^19^ By precisely manipulating the needle at the acupoint, a benign and minimally invasive form of stimulation can lead to the deformation of local connective tissue as well as the secretion of various bioactive molecules, including high mobility group box, adenosine, cyclic guanosine monophosphate, norepinephrine, calcium ion (Ca^2+^), and adenosine triphosphate (ATP).^18^, ^19^ In the study, we explored the role of TRPV1 at CV23 acupoint in EA to improve swallowing function. TRPV1 is an ion channel predominantly expressed in sensory neurons, eliciting an influx of cations upon activation.^20^ Through activating the TRPV1, acupuncture can generate local ATP release and Ca^2+^ wave propagation, resulting in signal transduction along the spinal cord.^16^ TRPV1 plays a crucial role in promoting nerve growth and regulating intracellular Ca^2+^ levels and has an effect on inflammatory reactions, neurotoxicity, and cell apoptosis.^21^, ^22^ TRPV1 has been demonstrated to play a fundamental role in the dynamic regulation of blood flow. Inhibiting TRPV1 activity has been found to result in increased coronary perfusion, decreased systemic blood pressure, and dilation of skeletal muscle arterioles.^23^ Furthermore, TRPV1 has been examined in the pharynx and closely associated areas.^24^ Activation of TRPV1 channels in swallowing‐related regions is evidenced to exert facilitative effects on the evocation of swallow reflex.^25^, ^26^ Therefore, we aimed to examine whether EA could regulate the swallowing function via TRPV1.

In the study, we observed an increase in blood perfusion and an activation of neuronal activity in the S1 following EA‐CV23. Subsequent injection of lidocaine at CV23 blocked these changes in blood perfusion and neuronal activity induced by EA treatment. Furthermore, the results showed an upregulation of TRPV1 near CV23 after EA treatment. Notably, the blood perfusion at CV23 but not in the S1 was reduced in the mice with downregulation of TRPV1 expression/function, while there was no change of neuronal activity in the S1, and similar results were observed in PSD mice.

## 2 MATERIALS AND METHODS

### 2.1 Animals

Male C57/BL6J mice and TRPV1‐KO mice aged 2–3 months were employed in all experimental procedures. C57 mice were sourced from the Animal Laboratory Center at Guangzhou University of Chinese Medicine, while TRPV1‐KO mice were purchased from Gene & Peace Co Ltd (Yangzhou, China). The animals had free access to food and water and were kept in temperature‐controlled rooms with a 12‐h light/dark cycle. The experimental procedures were performed in accordance with the National Institutes of Health Guide for the Care and Use of Laboratory Animals and were approved by the Committee for Care and Use of Research Animals of Guangzhou University of Chinese Medicine (No. 20170303). The experiments are reported following the ARRIVE guidelines (Animal Research: Reporting in vivo Experiments).

### 2.2 PSD modeling

As described previously,^11^ 1.5% rose bengal was administered intraperitoneally at a dose of 100 mg/kg. After about 10 min, a laser beam (532 nm) with a diameter of 1 mm was directed toward the right M1 cortex (AP: −0.12 mm, ML: −1.03 mm, DV: −1.10 mm) for 7 min (Figure S5A). For the Sham group, the same injection of rose bengal was administered without illumination.

### 2.3 Laser speckle contrast imaging

As described previously,^11^ mice were anesthetized and positioned on a stereotaxic apparatus prior to making a paramedian incision to access the skull bone to detect the blood perfusion in the M1/S1. Mice with anesthesia were laid supine and secured to a board following fur removal around the mylohyoid region. The laser was carefully centered near the CV23 acupoint (area: 4 mm^2^) for a recording period of 2 min. The variation of blood perfusion was analyzed with the analysis software PIMsoft.

### 2.4 EA procedure

The stimulating parameters included an intensity of 1 mA, a continuous frequency of 2 Hz, and a treatment duration of 15 min.^11^ The ham groups were anesthetized with isoflurane without EA stimulation. The Sham‐EA group received EA treatment for 15 min once at the sham acupoint (in the tail). The EA group was subjected to a single 15‐min session of EA‐CV23.

### 2.5 Water consumption test

As described previously,^11^ during the water test, each mouse was housed individually. The ultimate water consumption of each mouse was determined by subtracting the residual volume of water from the initial volume after the 24‐h period had elapsed.

### 2.6 Immunofluorescent staining

Mice were deeply anesthetized with 1.25% tribromoethanol (i.p) and perfused transcardially with 25 mL of saline, followed by 25 mL of 4% paraformaldehyde (PFA) to fix the brain tissue. The entire brain and tissue surrounding the CV23 were excised and post‐fixed overnight with 4% PFA. The specimens were dehydrated through a series of immersion steps in 15% and 30% sucrose solutions before being embedded in the optimal cutting temperature (OCT) compound. Using a freezing microtome, brain specimens were sectioned into segments of 40 μm, and tissue was sectioned into segments of 20 μm. The sections were obstructed at 37°C for 1.5 h using a blocking mixture that consisted of 1% BSA and 0.3% Triton X‐100. To examine the expression of c‐Fos, TRPV1, CGRP, and TH, the sections were incubated overnight with primary antibodies (rabbit anti‐cFos: Cell Signaling Technology, 2250, 1:500, mouse anti‐TRPV1: Abcam, ab203103, 1:500; rabbit anti‐CGRP: Cell Signaling Technology, 14959S, 1:500; rabbit anti‐tyrosine hydroxylase: Abcam, ab112,1:500).

The brain sections were incubated with the second antibody, anti‐mouse‐conjugated AlexaFluor488 (Life, A21202, 1:500), anti‐rabbit‐conjugated AlexaFluor488 (Invitrogen, A11034, 1:500), and anti‐rabbit‐conjugated AlexaFluor594 (Life Technologies, a21207,1:500) for 1 h at 37°C.

The sections underwent nuclear staining using a DAPI solution at a concentration of 1 μg/5 mL for a duration of 5–10 min. To visualize the samples at 20X or 40X magnification, a confocal microscope provided by Nikon in Japan was utilized. The images were analyzed by an operator who was blinded to the group allocation, using ImageJ (1.52a) analysis software.

### 2.7 Fiber photometry recording

The administration of the AAV2/9‐CaMKIIα‐GCaMP6s virus (250 nL) into the M1 (AP: −0.12 mm, ML:1.03 mm, DV: −1.10 mm) or the S1 (AP: 0.86 mm, ML: −3.0 mm, DV: −2.4 mm) was performed with a precisely controlled rate of 30 nL/min using an Ultra Micropump. Then, an optical fiber (230 mm OD, NA = 0.37, 3 mm length) was implanted into the M1 or S1. In order to record the Ca^2+^ fluorescent signals before (pre, 5 min), during (EA, 15 min), and after (post, 15 min) EA treatment, we employed the fiber photometry system. Afterward, the raw data were delicately exported to MATLAB for further, thorough analysis. Fluorescence signals were computed for a duration of 1 min during various conditions. The fluorescence was derived by calculating *Z*‐score = *V*_siganl −_ *F*_0_/σ*F*, σ*F* = STD (*V*_basal_), where *F*_0_ was determined by averaging the baseline fluorescence signal during the initial 3 s $F_{0}=\bar{V_{\text{basal}}}$.

### 2.8 In vivo EMG recording

To capture the electromyographic (EMG) signals, the mice were secured onto a mouse adaptor while being awake and positioned in a supine manner as described previously.^11^ The delivery of 20 μL of water was executed with a micro‐injection pump at a consistent rate of 2 μL/s. Spike2 software was utilized to elicit and record the EMG function of the mylohyoid muscle upon water delivery. Finally, to quantify the swallowing function following water delivery, the area under the curve (AUC) of the EMG signal was computed over a selected 10‐s time window.

### 2.9 RNA‐sequencing analysis

Muscle biopsy specimens surrounding CV23 were collected from both the Sham and EA groups, and the total RNA amounts and integrity were assessed with the RNA Nano 6000 Assay Kit, utilizing the Bioanalyzer 2100 system. To prepare libraries for sequencing, Novogene Bioinformatics Technology Co. conducted transcriptome sequencing. Differential expression analysis of the two groups/conditions (with two biological replicates per condition) was carried out using the DESeq2 R package (1.20.0). To control the false discovery rate, *p*‐values were adjusted with the approach by Benjamini and Hochberg. The criterion for defining significantly differential expression was set at Padj < 0.05 and |log_2_(foldchange)| > 1, respectively.

### 2.10 Enzyme‐linked immunosorbent assay (ELISA)

The tissue samples were homogenized in ice‐cold (−20°C) and the lysates' protein concentrations were assessed using the BCA method (Pierce), with efforts made to ensure equalization of the total protein content between samples. The levels of TRPV1 were quantitatively analyzed via an ELISA kit (R&D Systems) according to the vendor's prescribed methodology. To measure the relative levels of TRPV1 in the muscle tissue derived from the various experimental groups, an ELISA kit was utilized in strict accordance with the manufacturer's guidelines.

### 2.11 Data analysis and statistical tests

All statistical analyses were performed using the GraphPad Prism (version 7.0). The data were presented as mean ± standard deviation (SD) with error bars in the graphs denoting the SD. The normality of data was examined using the Shapiro‐Wilk normality test. Normally distributed data were analyzed using a two‐tailed Student's *t*‐test (unpaired) and one‐way ANOVA with Bonferroni post hoc test. Variance analysis of random block and non‐normally distributed data were analyzed by using Mann‐Whitney *U* test and Kruskal–Wallis with Tukey's post hoc test. In addition, Pearson correlation test was used to analyze the correlation between the two variables. Sigmaplot (version 14.0) software and BioRender.com platform were used for data visualization. Significance was considered at *p* < 0.05 (*), 0.01(**), or 0.001(***).

## 3 RESULTS

### 3.1 The EA enhanced the blood perfusion and neuronal activity in the S1 and this enhancement was dampened by local injection of lidocaine at CV23

**FIGURE 1:**
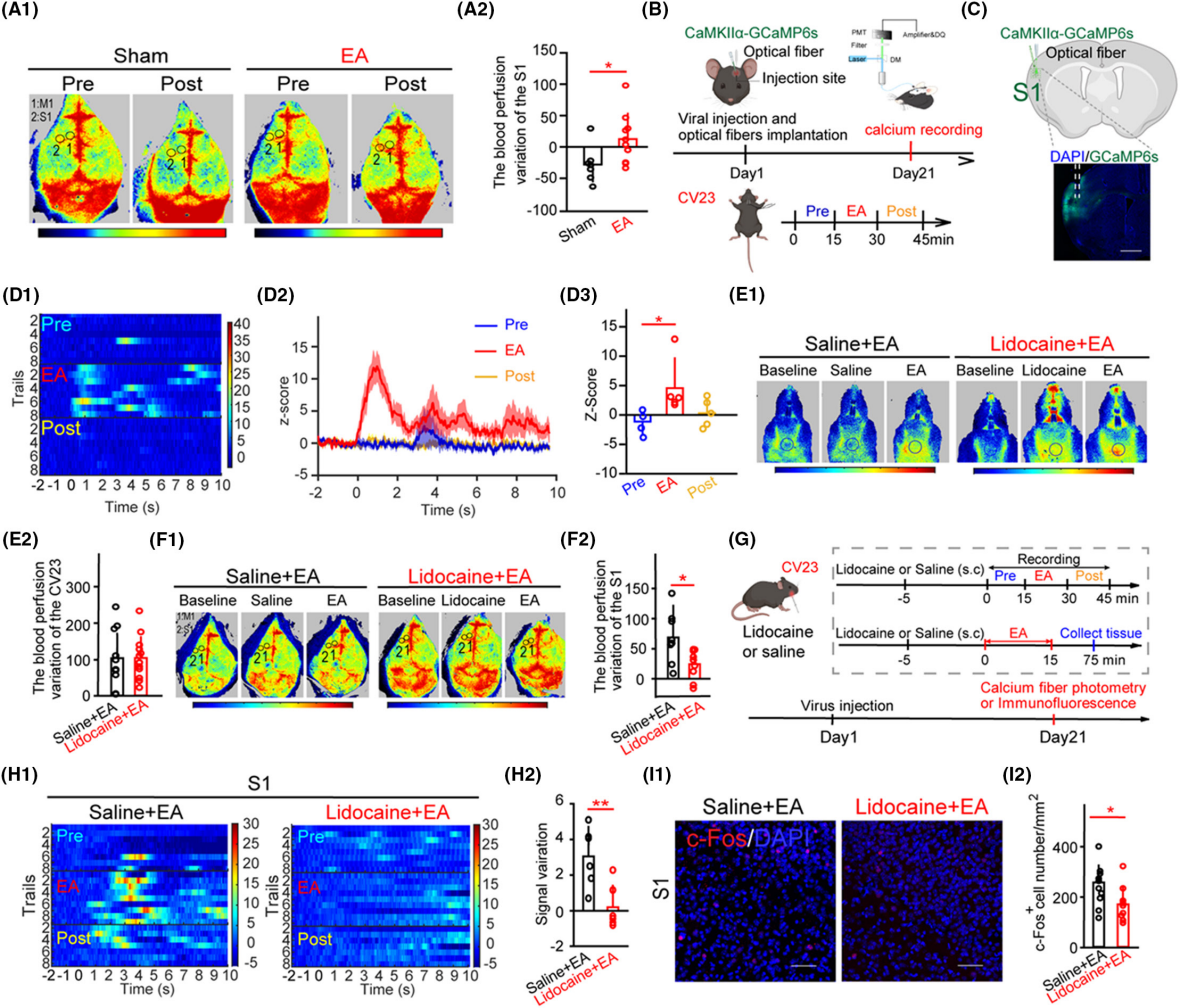
Lidocaine reduced the blood perfusion variation and neural activity of the S1 that increased by EA. (A) LSCI showed before (pre) and after EA treatment (post) in wild‐type mice. Sham group: lack of EA treatment, EA group: EA‐CV23. Blood perfusion was visualized as a two‐dimensional color‐coded map of blood flow, with red indicating high perfusion and blue indicating low perfusion. The region of interest (ROI) was defined as the area within which the mean cortical perfusion was calculated (A1). Time: 120 s, target 1: M1, target 2: S1, target area = 0.5 mm^2^. The blood perfusion variation was calculated as the difference between the blood perfusion in the post‐ and pre‐conditions. The results demonstrated a significant increase in blood perfusion variation in the S1 of the EA group compared to that in the Sham group (A2). Two‐tailed Student's unpaired *t* test, *n* = 7–10, *p < 0.05. (B) Schematic drawing of experimental protocol for fiber optic calcium recording. (C) Expression of CaMKIIα‐GCaMP6s in the S1 neurons. Dashed white lines indicate the position of optical insertion. Scale bars, 1000 μm. (D) A heatmap of individual GCaMP6s signals (D1) and an averaged GCaMP6s response curve (D2) were generated and aligned with the EA treatment to illustrate the kinetics of GCaMP6s signals from the CaMKIIα neurons in the S1. The pre (blue), EA (red), and post (yellow) conditions were displayed. Quantitative analyses were performed to determine the average change in GCaMP6s fluorescence (Z‐score) before, during, and after EA treatment. It revealed a significant increase of Ca^2+^ signals in S1 neurons during the EA condition, while no significant changes were observed in the post‐condition (D3). Kruskal–Wallis with Tukey's post hoc test, *n* = 5 per group, *p < 0.05, EA vs pre. (E) LSCI showed the baseline, injected, and after EA treatment in the lower jaw from the saline group and lidocaine group (E1). Time:120 s, black area: CV23, target area = 4 mm^2^. The results showed no significant difference in blood perfusion near CV23 between the lidocaine + EA group and the saline + EA group at the CV23 (E2). *N* = 10–13 mice. Two‐tailed Student's unpaired *t* test, *n* = 10–13, *p* > 0.05. (F) LSCI showed the baseline, injected, and post‐EA treatment blood perfusion in the brain of both saline + EA and lidocaine + EA groups (F1). Time: 120 s, target 1: M1, target 2: S1, target area = 0.5 mm^2^. The data showed a reduction in blood perfusion variation in the S1 of the lidocaine + EA group compared to that in the saline + EA group (F2). Two‐tailed Student's unpaired *t* test, *n* = 8–9, *p < 0.05. (G) Experimental design and timeline of the EA experiments. (H) A representative heatmap was constructed to demonstrate the relative change of Ca^2+^ signals in the S1 during pre, EA, and post conditions in both the saline + EA and lidocaine + EA groups (H1). The signal variation was decreased in the lidocaine + EA group compared to that in the saline + EA group. The signal variation was calculated by the signal in the EA conditions minus the signal in the pre‐conditions (H2). Two‐tailed Student's unpaired *t*‐test, *n* = 6 per group, **p < 0.01. (I) Representative images of c‐Fos immunofluorescence in the S1 following EA treatment after subcutaneous injection of saline or lidocaine in the CV23 (I1). Scale bar, 100 μm. The density of c‐Fos protein in the S1 following EA treatment was reduced in the lidocaine + EA group compared to that in the saline + EA group (I2). Two‐tailed Student's unpaired *t*‐test, *n* (slices) = 10–11, *p < 0.05. Data are shown as mean ± SD.

Sensory information strongly influences motor coordination,^27^ and M1 receives somatosensory input predominantly via S1.^14^ Consistent with previous studies,^8^, ^11^ elevated blood perfusion changes were observed in the M1 in the EA group compared to that in the Sham group (Figure S1A). The variation in the S1 was increased in the EA group compared to that in the Sham group (Figure 1A; *p* < 0.05). To exclude the potential possibility of difference between two hemispheres brain regions, the blood perfusion of M1 or S1 between hemispheres was detected, and the results showed no difference (Figure S1B). To investigate the influence of EA on neuronal activity in the S1, we performed fiber calcium recording (Figure 1B–D). It was observed that total integrated Ca^2+^ activities were significantly elevated during EA‐CV23 compared to that in the pre‐EA treatment, and the activities returned to the level of pre‐EA treatment in the post‐EA 15 min (Figure 1D3; *p* < 0.05), and there was no obvious change with CaMKIIα‐GFP virus injection (Figure S1D). The results of neuronal activity were similar in the M1 (Figure S1C,E). Therefore, EA‐CV23 increased the blood perfusion and neuronal activity of S1 in C57 mice.

The blood perfusion near CV23 was increased by EA‐CV23 rather than non‐acupoint (in the tail) (Figure S2A; *p* < 0.05). To determine the peripheral nerve impulses at CV23 are important for EA's effects, we first blocked the neuronal transmission by local application of lidocaine (a sodium channel blocker, 0.5% in 100 μL saline).^28^ The variation at the CV23, M1, and S1 was evaluated, respectively, and it was found no difference in the lidocaine‐injected and saline‐injected mice (Figure S2B). The blood perfusion variation of the S1 in the lidocaine + EA group was significantly decreased compared to that in the saline + EA group (Figure 1F; *p* < 0.05), but no difference in blood perfusion variation at CV23 (Figure 1E). We then asked whether this blockade by lidocaine was critical for the EA's effect on neuronal activity in the cortex (Figure 1G). It showed that the change of neuronal activity in the S1 was significantly weakened in the lidocaine + EA group compared to that in the saline + EA group (Figure 1H; *p* < 0.01). In addition, the density of c‐Fos in the S1 was also reduced (Figure 1I; *p* < 0.05). The M1 showed similar results that the blood perfusion variation and neuronal activation were weakened (Figure S2C–E). These results together suggested that local neurotransmission at CV23 was essential for EA‐mediated signal to the S1.

### 3.2 EA upregulated the expression of TRPV1 in the tissue near the CV23 acupoint

**FIGURE 2:**
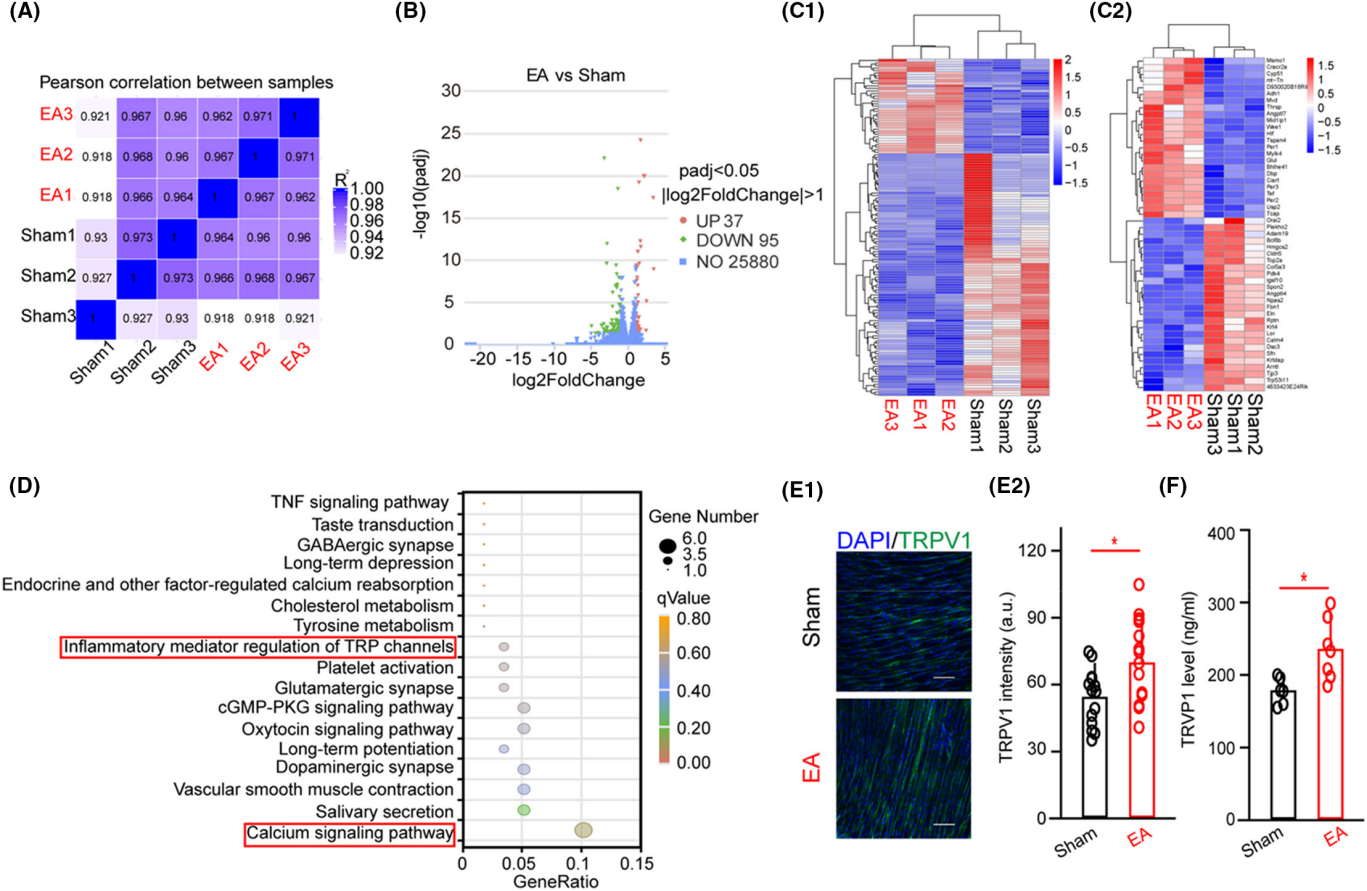
The expression of TRPV1 at CV23 was upregulated by EA. (A) A heatmap was generated to depict the Pearson correlation coefficient between the samples of the Sham and EA groups. The EA sample was obtained from the muscle surrounding CV23 with EA treatment, while the Sham sample was not subjected to EA treatment. (B) A volcano plot was constructed to visualize the differential regulation of gene expression between the Sham and EA groups, as determined by RNA‐Seq analysis. These upregulated and downregulated genes were visualized in red and green, respectively, while the values were expressed as the logarithmic base 2 of tag counts. (C) Hierarchical clustering of the RNA‐seq analysis results was performed to visualize all the genes that were significantly differentially expressed (C1), as well as the representative top 50 genes that exhibited differential expression (C2). (D) The KEGG pathway enrichment analysis was performed to evaluate the targets linked to EA ChIP‐seq peaks. (E) Representative images of immunofluorescent staining of TRPV1 (green) at the CV23 in the Sham and EA groups (E1). Scale bar, 100 μm. It showed a marked elevation in the intensity of TRPV1 immunopositive neurons at the CV23 in the EA group, as compared to that in the Sham group (E2). Two‐tailed Student's unpaired *t*‐test, *n* (slices) = 14–15, *p < 0.05. (F) It showed that the level of TRVP1 was increased by EA compared to that in the Sham group. Two‐tailed Student's unpaired *t*‐test, *n* = 6–7, *p < 0.05. Data are shown as mean ± SD.

To clarify how EA‐mediated signal to the S1, we performed gene expression profiling at CV23. The result from the mRNA sequence was reliable and the sample selection was reasonable (Figure 2A). Out of a total of 132 differentially expressed genes (DEGs), 37 gene transcripts exhibited upregulation, whereas 95 gene transcripts demonstrated downregulation in the EA group relative to the Sham group (Figure 2B). The altered representative genes and the representative top 50 most significant genes with altered expression are shown in Figure 2C. In addition, it showed that calcium signaling pathway, salivary secretion, the inflammatory mediator of TRP channels, vascular smooth muscle contraction, and tyrosine metabolism related to swallowing, neuronal activity, and blood perfusion (Figure 2D and Figure S3A). The calcium signaling pathway was the most significantly enriched pathway. Transient receptor potential vanilloid 1 (TRPV1) is a calcium channel mediating pain sensation,^29^ and is considered to be an “acupuncture‐responding channel”.^16^ To verify that EA influences the expression of TRPV1, we then performed immunofluorescence (Figure 2E) and ELISA (Figure 2F) after EA‐CV23. The expression of TRPV1 was increased by EA‐CV23 (Figure 2E,F; *p* < 0.05). We further found that anesthesia had no effect on TRPV1's expression (Figure S3B,C). Therefore, these results suggested that EA‐CV23 could activate the expression of TRPV1 at the acupoint.

### 3.3 Silencing of TRPV1 did not prevent the EA‐mediated effect on blood perfusion or neuronal activity in the S1, but altering the blood perfusion at the CV23

**FIGURE 3:**
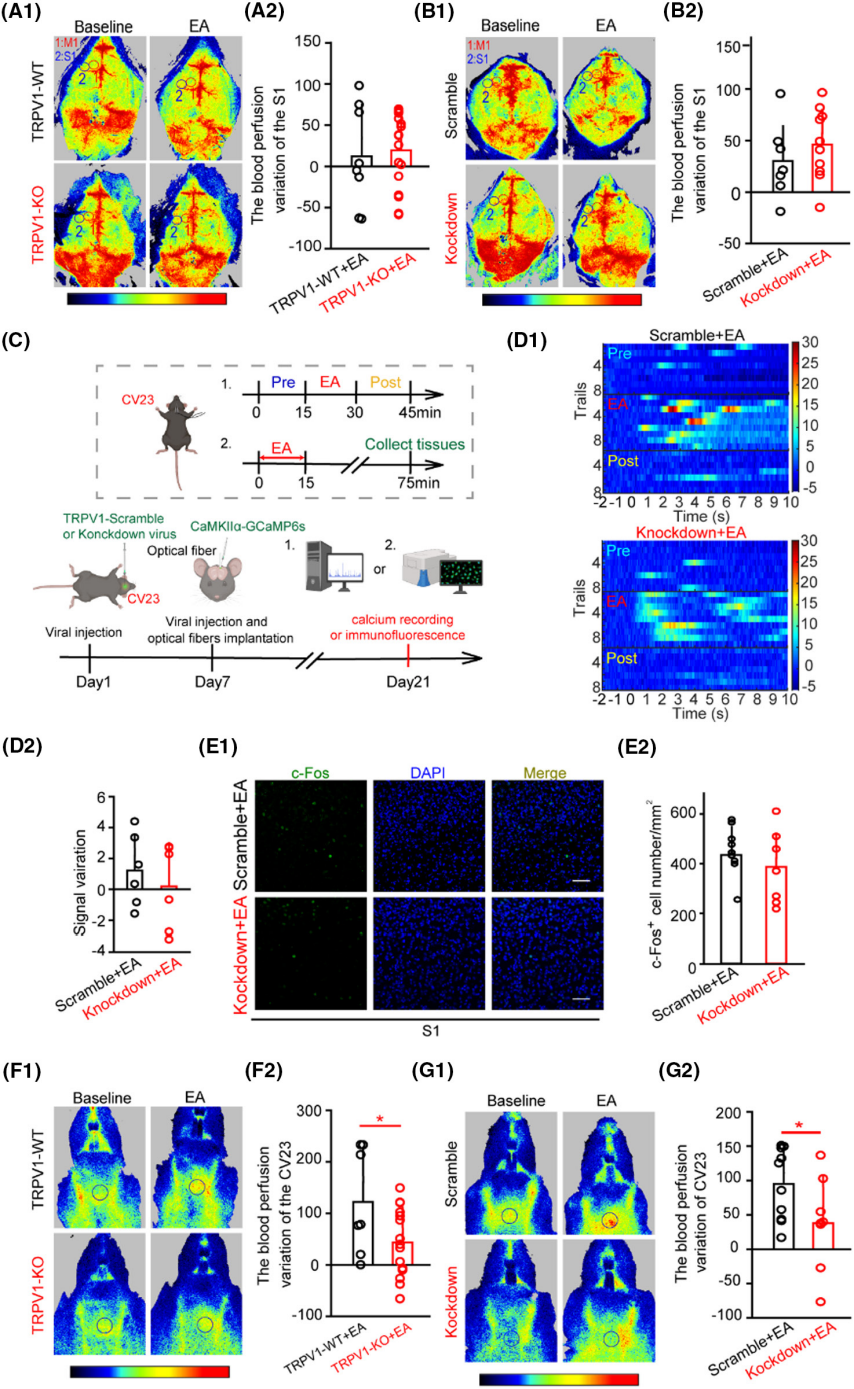
Silencing of TRPV1 decreased the blood perfusion variation at CV23 but did not affect the neuronal activity and blood perfusion variation in the S1. (A) Representative LSCI showed the blood perfusion of the brain in baseline, EA conditions from TRPV1‐WT and TRPV1‐KO groups. Time: 120 s, target 1: M1, target 2: S1, target area = 0.5 mm^2^ (A1). A bar graph revealed no change in blood perfusion variation of the S1 after EA treatment in the TRPV1‐KO group compared to that in the TRPV1‐WT group (A2). Mann‐Whitney *U* test, *n* = 8–17, *p* > 0.05. (B) Representative LSCI showed the blood perfusion of the brain in baseline, EA conditions from TRPV1‐scramble and TRPV1‐knockdown groups (B1). Time: 120 s, target 1: M1, target 2: S1, target area = 0.5 mm^2^. A bar graph revealed no change in blood perfusion variation of S1 after EA treatment in the TRPV1‐knockdown group compared to that in the TRPV1‐scramble group (B2). Two‐tailed Student's unpaired *t*‐test, *n* = 7–10, *p >* 0.05. (C) Schematic illustration of the experimental design. (D) Representative heatmap of CaMKIIα neurons showed the relative change of GCaMP6s fluorescence in the S1 during pre, EA, and post conditions in the knockdown + EA group and the scramble + EA group (D1). Quantification of the average amplitude of decreased signals in the knockdown + EA group compared to that in the scramble + EA group (D2). Two‐tailed Student's unpaired *t*‐test, *n* = 6 per group, *p >* 0.05. (E) Representative images of c‐Fos immunofluorescence in the S1 following EA treatment after intramuscular injection of scramble or knockdown virus at the CV23 (E1). Scale bar, 100 μm. A bar graph showed that the density of c‐Fos protein in the S1 following EA treatment was not changed in the knockdown + EA group compared to that in the scramble + EA group (E2). Two‐tailed Student's unpaired *t*‐test, *n* = 7–9, *p >* 0.05. (F) Representative LSCI showed the blood perfusion of the lower jaw in baseline, EA conditions from TRPV1‐WT and TRPV1‐KO groups (F1). Time: 120 s, black area: CV23, target area = 4 mm^2^. A bar graph revealed a decrease in blood perfusion of CV23 after EA treatment in the TRPV1‐KO group compared to that in the TRPV1‐WT group (F2). Two‐tailed Student's unpaired *t*‐test, *n* = 7–15, *p < 0.05. (G) Representative LSCI showed the blood perfusion of the lower jaw in baseline, EA conditions from TRPV1‐scramble and TRPV1‐knockdown groups (G1). Time: 120 s, black area: CV23, target area = 4 mm^2^. A bar graph revealed a decrease of blood perfusion variation at CV23 after EA treatment in the TRPV1‐knockdown group compared to that in the TRPV1‐scramble group (G2). Two‐tailed Student's unpaired *t*‐test, *n* = 8–9, *p < 0.05. Data are shown as mean ± SD.

To confirm the role of TRPV1 in the EA‐mediated improvement of swallowing function, we firstly detected the blood perfusion variation in the S1 from the TRPV1‐KO and TRPV1‐WT groups. The results showed that TRPV1‐KO mice did not affect the variation in the S1 induced by EA‐CV23 (Figure 3A). We further injected TRPV1 shRNA (TRPV1‐knockdown) virus into the CV23 to locally downregulate the TRPV1 expression/function. RT‐PCR studies revealed that TRPV1 expression at the CV23 was attenuated in TRPV1‐knockdown group (Figure S4A; *p* < 0.05). Consistent with the results obtained from the TRPV1‐KO mice, there was no difference in blood perfusion variation in the S1 between groups (Figure 3B). Therefore, silencing of TRPV1 at CV23 did not prevent the EA‐mediated increase of blood perfusion in the S1. Next, we further explored whether TRPV1 at the CV23 was involved in the neuronal activity of the S1 (Figure 3C). It showed no significant change in the TRPV1‐knockdown group compared to that in the TRPV1‐scramble group (Figure 3D,E). Moreover, the similar results were observed in the M1 (Figure S4B–E). Taken together, these findings indicated that TRPV1 did not affect the EA‐mediated increase of blood perfusion and neuronal activation in the S1.

In the study, the results showed that TRPV1 had no effect on the S1 induced by EA‐CV23, but whether it had any effect on local blood perfusion needs to be further tested. Previous studies showed that the blood perfusion variation at CV23 was increased by EA‐CV23 (Figure S2A).^8^ However, the blood perfusion variation at the CV23 was reduced in the TRPV1‐KO mice (Figure 3F; *p* < 0.05). Besides, the similar results were observed in the TRPV1‐kncokdown mice (Figure 3G; *p* < 0.05). Then, a typical TRPV1 antagonist, AMG9810 (20 μL, 10 nmol),^30^ was intramuscular (i.m) injected into the CV23 to block the TRPV1 function pharmacologically, and the blood perfusion variation was also decreased (Figure S4F; *p* < 0.05). Similar results were observed after intraperitoneal (i.p) injection of AMG9810 (1 mg/kg) (Figure S4G; *p* < 0.05). Therefore, TRPV1 at CV23 acupoint played an important role in the local blood perfusion increased by EA.

### 3.4 TRPV1 involved the EA‐CV23‐mediated increase of blood perfusion in the tissue near the acupoint and improvements in swallowing function in PSD mice

**FIGURE 4:**
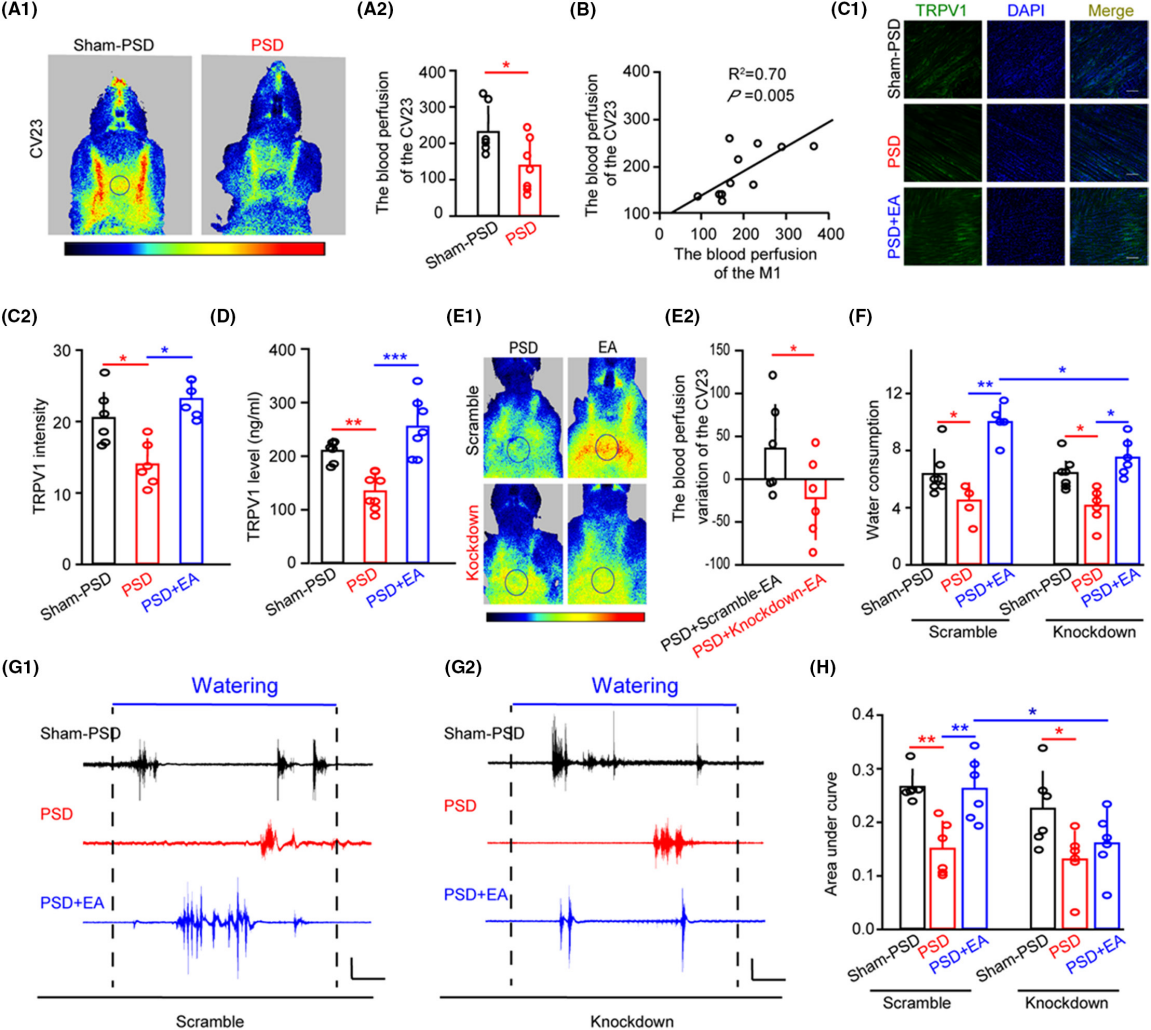
EA‐CV23 increased local blood flow by TRPV1 to improve swallowing function in PSD mice. (A) Representative LSCI of the lower jaw in the Sham‐PSD and the PSD group (A1). Time: 120 s, black area: CV23, target area = 4 mm^2^. A bar graph showed the blood perfusion of the CV23 in the PSD group was lower than that in the Sham‐PSD group (A2). Two‐tailed Student's unpaired *t*‐test, *n* = 7 per group, *p < 0.05. (B) Positive correlation between the perfusion of the M1 and the CV23 analyzed by laser speckle. A significantly positive statistical correlation was found between the groups. *R* ^2^ = 0.70, *p* < 0.01. (C) Representative immunofluorescence images of TRPV1 were performed at the CV23 from Sham‐PSD, PSD, and PSD + EA groups (C1). Sections were stained with anti‐TRPV1 (green) and counterstained with DAPI (blue). Scar Bars, 100 μm. The intensity of TRPV1 was significantly reduced by stroke induction and this impairment could be recovered by EA treatment (C2). one‐way ANOVA with Bonferroni post‐hoc test, *n* = 6 per group, PSD vs Sham‐PSD: *p < 0.05, PSD + EA vs PSD:*p < 0.05. (D) The level of TRPV1 was reduced by stroke induction, and the phenomenon was recovered by EA treatment. Kruskal‐Wallis with Tukey's post hoc test, *n* = 7–8, PSD vs Sham‐PSD: **p < 0.01, PSD + EA vs PSD: ***p < 0.001. (E) Representative LSCI showed the blood perfusion of the lower jaw in PSD and PSD + EA conditions from TRPV1‐scramble and TRPV1‐knockdown groups (E1). Time: 120 s, blue area: CV23, target area = 4 mm^2^. A bar graph revealed a decrease in blood perfusion of CV23 after EA treatment with PSD in the TRPV1‐knockdown group compared to that in the TRPV1‐scramble group (E2). Two‐tailed Student's unpaired *t*‐test, *n* = 6 per group, *t* = 2.714, *p < 0.05. (F) The water consumption was reduced by stroke induction and increased by EA treatment in the TRPV1‐scramble group and the TRPV1‐knockdown group, and the water consumption was decreased in the TRPV1‐knockdown  group compared to that in the TRPV1‐scramble group in the PSD + EA conditions. One‐way ANOVA with Bonferroni post‐hoc test, *n* = 6–7 per group, scramble: PSD vs Sham‐PSD: *p < 0.05, PSD + EA vs PSD: **p < 0.01. Knockdown: PSD vs Sham‐PSD: *p < 0.05, PSD + EA vs PSD: *p < 0.05, Two‐tailed Student's unpaired *t*‐test, scramble‐PSD + EA vs knockdown‐PSD + EA, *p < 0.05. (G) Example trace of EMG response to watering stimulation in the Sham‐PSD, PSD, and PSD + EA mice from the TRPV1‐scramble group (G1) and TRPV1‐knockdown group (G2). Scale bars, time = 2 s, bin = 0.1 mv. (H) The EMG response was decreased by stroke induction and recovered by EA treatment in the TRPV1‐scramble group. The EMG response was decreased by stroke induction but not recovered by EA treatment in the TRPV1‐knockdowngroup, and the EMG response was decreased in the TRPV1‐knockdown + EA group compared to that in the TRPV1‐scramble + EA group in the PSD + EA conditions. one‐way ANOVA with Bonferroni post‐hoc test, *n* = 6 per group, scramble: PSD vs Sham‐PSD: **p < 0.01, PSD + EA vs PSD: **p < 0.01, knockdown: PSD vs Sham‐PSD: *p < 0.05, PSD + EA vs PSD: *p* > 0.05. Two‐tailed Student's unpaired *t*‐test, scramble‐PSD + EA vs knockdown‐PSD + EA, *p < 0.05. Data are shown as mean ± SD.

To investigate the impact of TPRV1 on CV23 in the improvement of swallowing function, we proceeded to induce a PSD mouse model as previously established (Figure S5A).^11^ EMG response and water consumption (Figure S5B,C) were impaired in PSD mice, which was similar to previous studies.^8^ Furthermore, PSD mice resulted in a decrease in mean motion range ratio (MMRR) assessed by laryngoscopy (Figure S5D). Notably, the blood perfusion at M1 and CV23 was also reduced in the PSD mice (Figure 4A and Figure S5E), and the change of CV23 had a strong correlation with M1 (Figure 4B).

To investigate the role of TRPV1 at the CV23 in the PSD mice, we first examined the expression of TRPV1 by immunofluorescence and ELISA (Figure 4C,D). We found that the expression of TRPV1 was significantly lower in the PSD mice, but EA could reverse the decrease (Figure 4C,D). To determine whether TRPV1 involved the blood perfusion effects induced by EA treatment in PSD mice, we examined the blood perfusion variation at CV23. The result showed that the blood perfusion of CV23 in the TRPV1‐knockdown group after EA treatment was far lower than that in the TRPV1‐scramble group (Figure 4E; *p* < 0.05). Notably, the blood perfusion in the S1 was also reduced in the PSD mice compared to that in the Sham‐PSD mice (Figure S5E), which had a strong correlation with M1 (Figure S5F), but it showed no obvious difference in the M1 and the S1 between the TRPV1‐kncokdown and TRPV1‐scramble groups in the PSD mice with EA treatment (Figure S5G). Therefore, TRPV1 near CV23 acupoint played an important role in the local blood perfusion increased by EA in the PSD mice.

Then, we explored the role of TRPV1‐meditated EA‐CV23 in regulating the swallowing function in PSD mice. The stroke induction led to a significant reduction in water consumption in both the TRPV1‐scramble and TRPV1‐knockdown groups. However, we observed a recovery of water consumption in these groups following EA treatment. Notably, in the PSD + EA group, after knocking down TRPV1 at CV23, we observed a persistent decrease in water consumption (Figure 4F; *p* < 0.05; *p* < 0.01). Similarly, the results showed that the EMG response was impaired by stroke induction and restored by EA, but the EMG response was not recovered by EA treatment in the TRPV1‐knockdown group (Figure 4G,H; *p* < 0.05; *p* < 0.01). Altogether, EA restored the swallowing function through TRPV1 at CV23 in PSD mice. These results suggested that TRPV1 was involved in the EA‐mediated improvement of blood perfusion near CV23, which contributed to the EA‐CV23‐regulated swallowing function.

## 4 DISCUSSION

Our findings revealed that TRPV1 at CV23 could modulate EA‐mediated increase of local blood perfusion near CV23 without affecting the EA‐mediated enhancement of blood perfusion and neuronal activity in the S1. Our study suggested the importance of TRPV1 at the CV23 during the EA treatment for PSD rehabilitation.

The significance of acupuncture is attributed to the involvement of afferent nerve fibers and diverse receptors located at the acupoint, which have been extensively studied in recent years.^31^, ^32^ Activation of the M1‐mylohyoid pathway by EA‐CV23, as demonstrated in recent studies,^11^ has been shown to induce a notable increase in motor conduction velocity within the hypoglossal nerve.^8^ By transmitting sensory information pertaining to deglutition to the brainstem, these nerves actuate the swallowing reflex and facilitate the activation of the motor cortex.^11^ The precise involvement of sensory afferent nerves located near the CV23 acupoint in the therapeutic effects of EA remains to be elucidated. Our previous studies have found that a pivotal M1‐parabrachial nuclei (PBN)‐nucleus tractus solitary (NTS) neural network is integral in regulating the swallowing process.^11^ However, no studies have reported the afferent pathway of EA‐CV23 in the regulation of swallowing function. In this study, we provided some evidence of the role of  a local molecules near CV23 in the acupuncture stimulation transmitting to the cerebral cortex. However, to dig out the mechanism of EA treatment for PSD, the peripheral‐central afferent pathway of EA‐CV23 should be detected in the future.

Furthermore, it is plausible that the effect of acupuncture is linked to its modulation of neurotransmitters.^33^ EA‐CV23 elicits the secretion of peripheral neurotransmitters, notably substance P (SP), thereby modulating the excitability of local primary afferent sensory neurons.^8^ In the present study, we mainly probed the involvement of TRPV1 situated at CV23 in the modulation of swallowing function. Our results indicated that TRPV1‐mediated the local blood perfusion at CV23 enhanced by EA but had no effect on blood perfusion and neuronal activity in the S1. The impact of acupuncture treatment is not attributable to a singular factor, but rather the intricate interplay of multiple levels, systems, and factors.^31^ Downregulation of TRPV1 expression/function at CV23 did not affect the activity of neuronal activity in the cerebral cortex. Aside from TRPV1, diverse proteins have been implicated in the modulation of blood perfusion. EA intervention can regulate the expression of tyrosine hydroxylase (TH) and calcitonin gene‐related peptide (CGRP).^34^, ^35^ The results suggested the expression of CGRP/TH was higher near the acupoint after EA‐CV23 (Figure S6A,C), and blood perfusion was reduced after blockade of the CGRP or TH (Figure S6B,D). Therefore, the effect of EA associated with blood perfusion was influenced by multiple receptors, and the role of TH and CGRP in EA remains to be further explored. In addition, other ion channels such as TRPV2,^24^ TRPA1,^36^, ^37^ TRPM8,^26^, ^37^ and P2X3^38^ have been found to be present in the area near the pharynx and are closely related to swallowing. These neurons may respond to chemical, thermal, and mechanical stimuli during respiration and swallowing. In future studies, we plan to investigate the role of these ion channels in EA.

TRPV1 exhibits ubiquitous expression throughout the brain, encompassing key regions such as the hippocampus, cortex, cerebellum, olfactory bulb, mesencephalon, and hindbrain, with its activation exerting a discernible impact on the pathophysiological state of cerebral ischemia.^39^ Numerous recent investigations have intimated that TRPV1 is implicated in stroke pathophysiology, yet the particular mechanism eludes comprehension.^40^ Administration of a TRPV1 antagonist via intracerebroventricular injection 30 min anterior to the onset of ischemia abated neurological and motor deficits and reduced infarct size.^40^ In this study, we investigated the role of TRPV1 at CV23 in mediating the effects of EA in treating PSD mice. The role of TRPV1 in the brain remains unclear and warrants further exploration in future studies.

One limitation of this study is that it focused solely on the immediate effects of EA. Changes in blood perfusion and neuronal activity were detected immediately after EA, and the duration of EA's effects remains unknown. In future studies, we plan to set up several additional time points to observe the efficacy time of EA. However, the signal recorded by the fiber optic recording decays over time, so long‐term neuronal activity can be explored through in vivo electrophysiology. Additionally, the population Ca^2+^ activity was found to be strongly dependent on the level of anesthesia,^41^ which is a factor that can influence the activity of the recorded neurons. These are important considerations for future research on EA.

In this study, we have demonstrated that the regulation of blood perfusion and the improvement of swallowing function mediated by EA are associated with TRPV1 at CV23. Our findings provide valuable insights into the potential mechanism underlying EA treatment for improving swallowing function in PSD.

## AUTHOR CONTRIBUTIONS

Nenggui Xu and Lulu Yao designed and guaranteed the whole experiment studies. Si Yuan, Bo Qiu, Ying Liang, and Bing Deng carried out all the experiments. Jing Xu, Xiaorong Tang, Junshang Wu, Sheng Zhou, Zeli Li, Hongzhu Li, Qiuping Ye, Lin Wang, Shuai Cui, and Wei Yi contributed to discussion, Si Yuan, Bo Qiu, and Ying Liang drafted the manuscript. Si Yuan, Nenggui Xu, and Lulu Yao edited and revised the whole manuscript. All authors read and approved the final manuscript.

## FUNDING INFORMATION

This study is supported by the General Program of the National Natural Science Foundation of China (no: 82374573), by the Support Program for Chinese Medicine Innovation Teams and Talents of the State Administration of Traditional Chinese Medicine (no: ZYYCXTD‐C‐202004), by the Guangdong Provincial Key Area R&D Program “Modernization of Ling Nan Traditional Chinese Medicine” Key Special Project (no: 2020B1111100008), and by the Youth Program of the National Natural Science Foundation of China (no: 82004469).

## CONFLICT OF INTEREST STATEMENT

The authors declare no competing interests.

## Supporting information


Data S1:

Figure S1:

## Data Availability

The data obtained in this research are available from the corresponding author upon reasonable request.
