# Supplementary material for: Role of TRPV1 in electroacupuncture‐mediated signal to the primary sensory cortex during regulation of the swallowing function
Source: CNS Neurosci Ther. 2023 Sep 18;30(3):e14457. doi: 10.1111/cns.14457 (PMC10916430; doi:10.1111/cns.14457)
Supplement: Supplementary file 1 — Data S1: [file CNS-30-e14457-s002.docx]

**Drug administration**

AMG9810 was dissolved in DMSO and TWEEN 80. Mice were achieved by a single intramuscular injection (i.m., 20 μl, 10 nmol) or a single intraperitoneal injection (i.p., 1mg/kg). The same dose of normal DMSO was in the DMSO + EA group. HCGRP-(8-37) was dissolved in saline solution, and the mice were intramuscular (i.m.) injected with HCGRP-(8-37) at a dose of 20 μl (100 μg/kg) in the HCGRP-(8-37) + EA group. H-Tyr(3-I)-OH was dissolved in saline solution, and the equivalent of 10 mg/kg body weight of free H-Tyr(3-I)-OH was intramuscular (i.m.) administered in the H-Tyr(3-I)-OH + EA group. Chemicals were purchased from MedChemExpress (USA). 2% lidocaine was diluted into 0.5% with saline and injected subcutaneously (s.c) into acupoints (0.1ml). The same dose of sterile normal saline was in the saline+ EA group.

**Transoral Laryngoscopy**

In accordance with our established protocol and custom equipment, the mice underwent transoral endoscopy to assess craniofacial structure and function. To maintain light sedation and local limb movement only in response to toe pinch, the mice were anesthetized through intraperitoneal (i.p.) injection of tribromoethanol (1.25%, 125 mg/kg, USA) and restrained ventrally on a foam plate secured with black insulating tape. A cotton swab and gentle finger grip were utilized to retract the tongue, after which an endoscope was deftly inserted into the oral cavity, allowing for visualization of baseline vocal fold (VF) movement. Gradually advanced, the endoscope captured bilateral VF movement and was held steady within the larynx for roughly 60 seconds to capture the progression of spontaneous abduction and adduction motion, constituting the respective inspiratory and expiratory respiratory phases. These procedures were enacted both pre-and post-modeling, whereby VF movement was assessed as tracked bilaterally by two trained, blinded reviewers, with the mean motion range ratio (MMRR) later being calculated. To supplement these proceedings, the whole endoscopic procedure was filmed at 30 frames per second and stored as MP4 files.

**Quantitative real-time polymerase chain reaction (qPCR)**

The muscle tissue of CV23 from diverse groups was promptly excised and flash-frozen in liquid nitrogen. TRIzol Reagent (product ID 15596018, Ambion) was used for total RNA extraction. After treating with 800 µL TRIzol, the samples were homogenized on ice with an Ultrasonic Cell Disrupter System. Next, 200 µL Chloroform was added, resulting in vortexing and centrifugation at 12000 rpm for 15 min at 4 °C. Subsequently, 150 µL of the supernatant was carefully transferred to a new tube. RNA was precipitated by mixing with 500 µL isopropyl alcohol followed by a 10-minute incubation after being inverted for mixing. The resulting samples were centrifuged at 12000 rpm for 10 min at 4 °C, and the pellet was washed with 1 mL 75% ethanol and spun at 7500 rpm for 5 min at 4 °C. After air-drying, the pellet was resuspended in 20 µL RNA dse-free water and quantified using an ultra-micro spectrophotometer (ND-2000, Thermo Fisher Scientific). According to the instructions of the reverse transcription kit (product ID RR047A-2, Takara), cDNA synthesis was carried out. Then, the quantitative real‐time PCR was conducted using the ABI PRISM 7300 real‐time PCR system (A28108, Thermo Fisher Scientific) with the primers (TRPV1:5′-CATGCTGGTGTCTGTGGTACTGTAC-3′ and 5′-TCTGCTGGAATCCTCGGGTGTAG-3′).

**Product information**

| Bioanalyzer 2100 system | Agilent Technologies | USA |
| --- | --- | --- |
| BSA | Macklin | China |
| Confocal microscope | Nikon | Japan |
| DAPI | Sigma | USA |
| ELISA kit | MEIMIAN | China |
| Fiber photometry system | Thinker Tech | China |
| Freezing microtome | Thermo | USA |
| ImageJ | National Institutes of Health | USA |
| Laser beam | Laserware | China |
| Micro-injection pump | HARVARD | USA |
| OCT | Sakura Finetek | USA |
| Rose bengal | Sigma-Aldrich | USA |
| Stereotaxic apparatus | RWD | USA |
| Tribromoethanol | Sigma–Aldrich | USA |
| Triton X-100 | Biosharp | China |
| Ultra Micropump | RWD | USA |
