# Supplementary material for: Role of TRPV1 in electroacupuncture‐mediated signal to the primary sensory cortex during regulation of the swallowing function
Source: CNS Neurosci Ther. 2023 Sep 18;30(3):e14457. doi: 10.1111/cns.14457 (PMC10916430; doi:10.1111/cns.14457)
Supplement: Supplementary file 2 — Figure S1: [file CNS-30-e14457-s001.docx]

**Supplementary figure1**


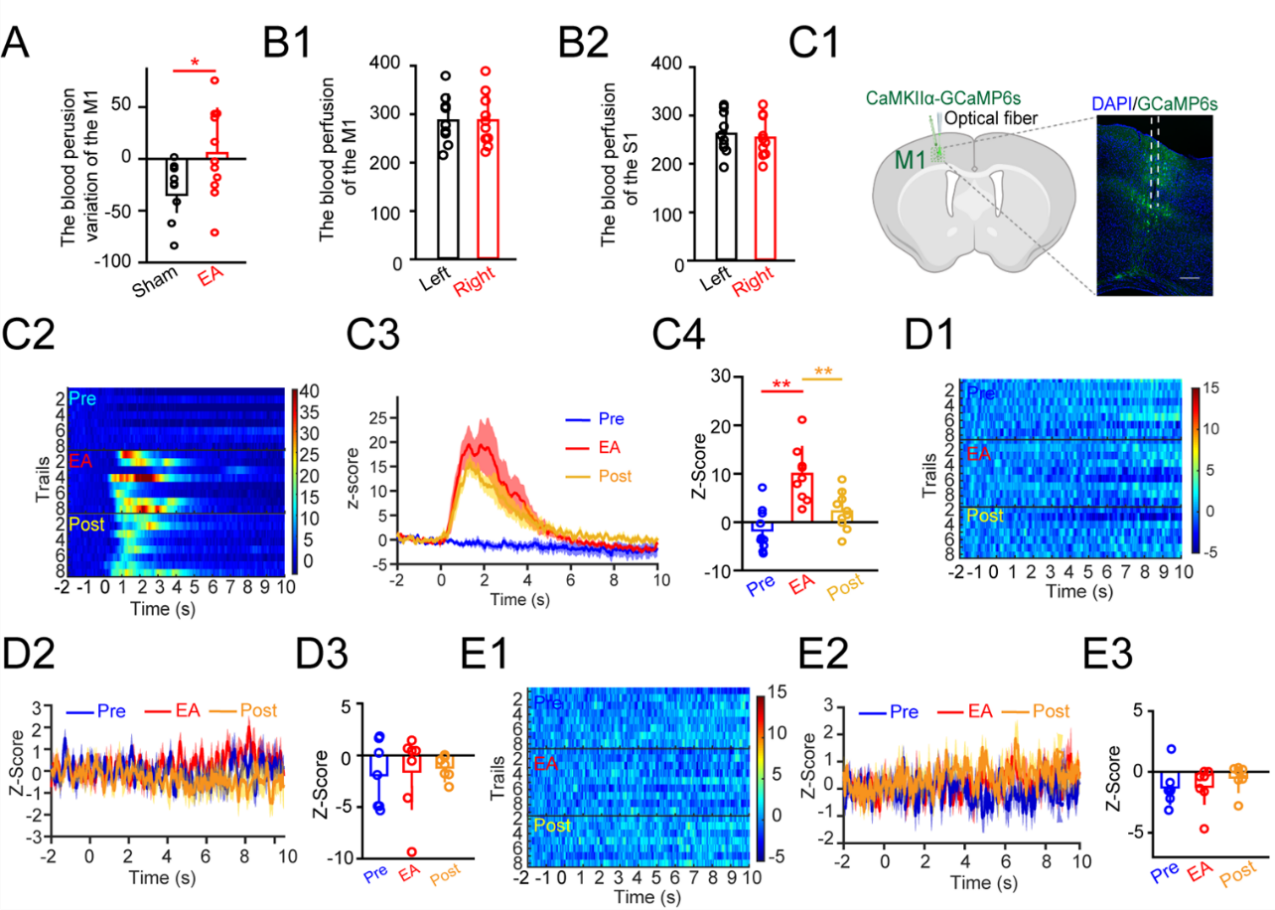


**Supplementary figure1:**

**(A)** A bar graph comparing the blood perfusion variation before (Pre) and after (Post) EA treatment in the M1 from the Sham and EA groups. The results showed that the blood perfusion variation in the M1 was significantly increased in the EA group. The blood perfusion variation was calculated by the blood perfusion in the Post minus that in the Pre. Two-tailed Student’s unpaired t-test, n=11 per group, **P* <0.05.

**(B**) A bar graph showed that there was no difference in the blood perfusion of the M1 **(B1)** and the S1 **(B2)** between the Left and Right hemispheres. n=10 per group.

**(C)** Expression of CaMKIIα-GCaMP6s in the M1 neurons **(C1)**. Dashed white lines indicate the position of optical inserts. Scale bars, 200 µm. A heatmap of individual GCaMP6s signals **(C2)** and an averaged GCaMP6s response curve **(C3)** aligned with the EA treatment, showing the kinetics of GCaMP6s signals in the M1. Pre (blue), EA (red), and Post (yellow) conditions were displayed. Quantitative analyses of average GCaMP6s fluorescence change (Z-score) before (Pre), during (EA), and after (Post) EA treatment **(C4)**. The results showed that Ca^2+^ signals in M1 neurons were increased in EA conditions, but the signals weren’t changed in the Post conditions. one-way ANOVA with Bonferroni post hoc test, n=10 per group, EA vs Pre: ***P* < 0.01, Post vs EA: ***P* < 0.01.

**(D)** Representative averaged GFP fluorescence dynamics **(D1)** and photometry traces **(D2)** of CaMKIIα -GFP neurons in the M1 during EA treatment in each session. Pre (blue), EA (red), and Post (yellow) conditions were displayed. Quantitative analyses of average GFP fluorescence change (Z-score) before (Pre), during (EA), and after (Post) EA treatment **(D3)**. The results showed that EA treatment didn’t evoke substantial changes in the GFP fluorescence of the M1 in the C57 mice injected with the AAV-CaMKIIα-GFP. n=6 per group.

**(E**) Representative averaged GFP fluorescence dynamics **(E1)** and photometry traces **(E2)** of CaMKIIα -GFP neurons in the S1 during EA treatment in each session. Pre (blue), EA (red), and Post (yellow) conditions are displayed. **(E3)** Quantitative analyses of average GFP fluorescence change (Z-score) before (Pre), during (EA), and after (Post) EA treatment. The results showed that EA treatment didn’t evoke substantial changes in the GFP fluorescence of the S1 in the C57 mice injected with the AAV-CaMKIIα-GFP. n=6 per group.

Data are shown as mean ± SD.

**Supplementary figure 2**


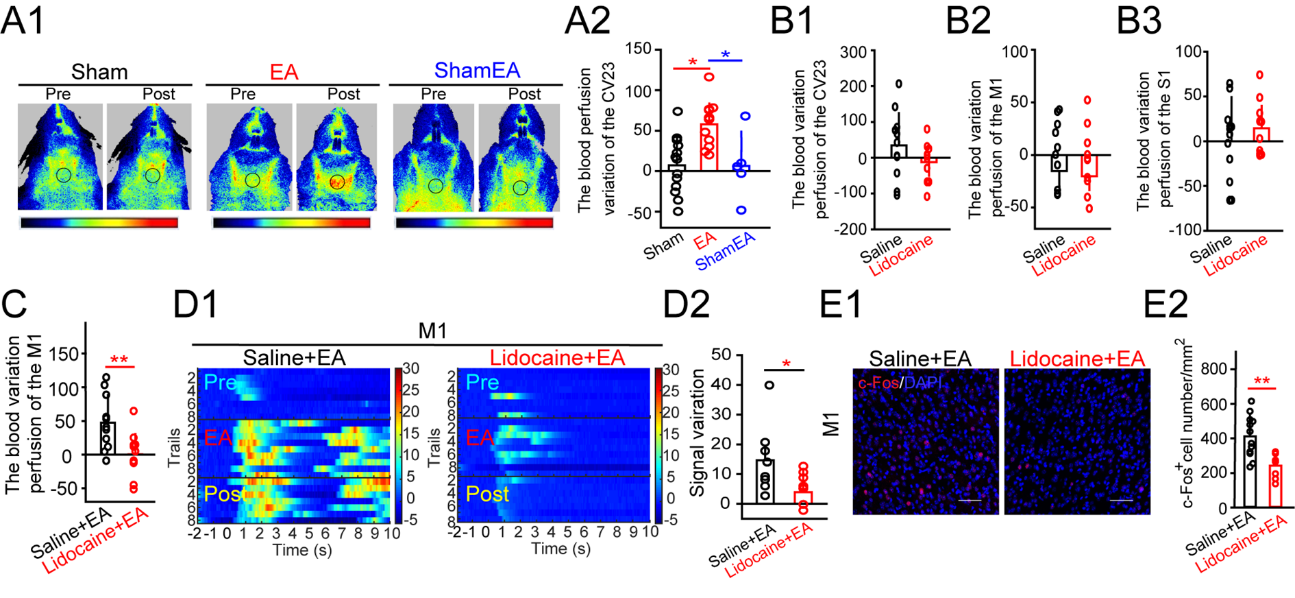


**Supplementary figure2:**

**(A)** LSCI showed before (Pre) and after EA treatment (Post) in the lower jaws from the Sham, EA, and Sham-EA groups **(A1)**. Time: 120 s, black area: CV23, target area = 4 mm^2^.A bar graph showed the blood perfusion variation of the CV23 from the Sham, EA, and Sham-EA groups **(A2)**. The results showed that the blood perfusion variation of the CV23 was increased in the EA group compared to that in the Sham group and the Sham-EA group, but there was no difference between the Sham group and the Sham-EA group. one-way ANOVA with Bonferroni post hoc test, n=7~12, EA vs Sham: **P* < 0.05, ShamEA vs EA: **P* < 0.05.

**(B)** A bar graph compared the blood perfusion variation in the CV23 **(B1)**, M1 **(B2)**, and S1 **(B3)** following intramuscular injection of Saline or Lidocaine at the CV23. The results showed that there was no difference among the groups. n=8~12.

**(C)** A bar graph showed the blood perfusion variation in the M1 before and after EA treatment in the Lidocaine and Saline group. The results showed that the blood perfusion variation of the M1 was reduced in the Lidocaine+ EA group. Two-tailed Student’s unpaired t-test, n=12 per group, ***P* <0.01.

**(D)** Representative heatmap of CaMKIIα^+^ neurons in the M1 of saline or lidocaine injection locally at the CV23 **(D1)**. It showed the relative change of fluorescence in the M1 during Pre, EA, and Post from the Saline+ EA group and Lidocaine+ EA group. Quantification of the average amplitude of decreased signals in the Lidocaine+ EA group compared to that in the Saline+ EA group **(D2)**. The signal variation was calculated by that the signal in the EA conditions minus that in the Pre conditions. Two-tailed Student’s unpaired t-test, n=9 per group, **P* <0.05.

**(E)** Representative images of c-Fos immunofluorescence in the M1 following EA treatment after subcutaneous injection of Saline or Lidocaine at the CV23 **(E1)**. Scale bars, 100 µm. A bar graph showed that a reduction in c-Fos protein density in the M1 subsequent to EA treatment was observed in the Lidocaine +EA cohort in comparison to the Saline +EA group **(E2)**. Two-tailed Student’s unpaired t-test, n (slices) =9~12, ***P* <0.01.

Data are shown as mean ± SD.

**Supplementary figure3**


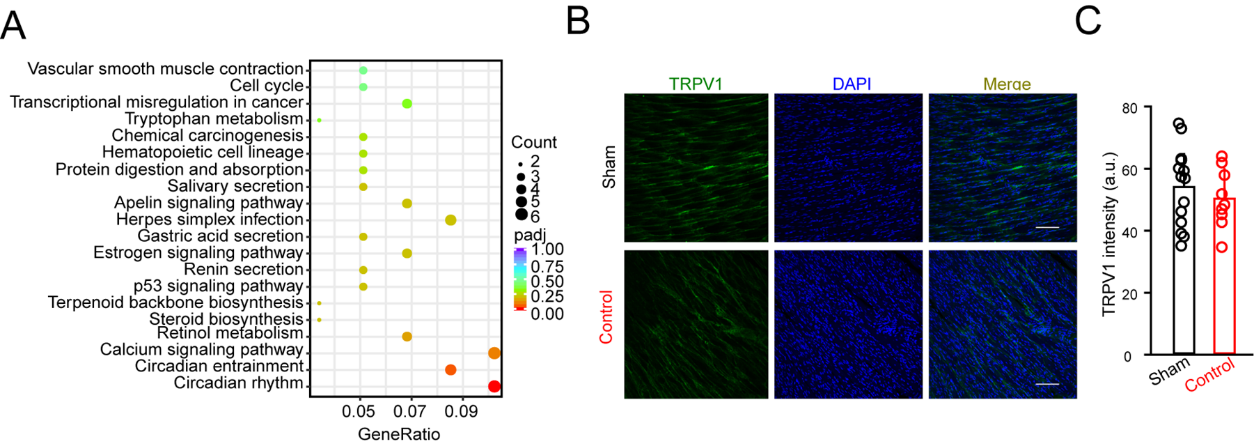


**Supplementary figure3:**

**(A)** The representative top 20 KEGG pathways enrichment analysis of targets associated with EA ChIP-seq peaks.

**(B)** Representative images of immunofluorescent staining of TRPV1 (green) in the Sham and Control groups. Scale bars, 100 µm.

**(C)** Quantification analysis showed that the intensity of TRPV1 wasn’t changed in the Sham group compared to that in the Control group. n (slices) = 9 ~ 14.

Data are shown as mean ± SD.

**Supplementary figure4**


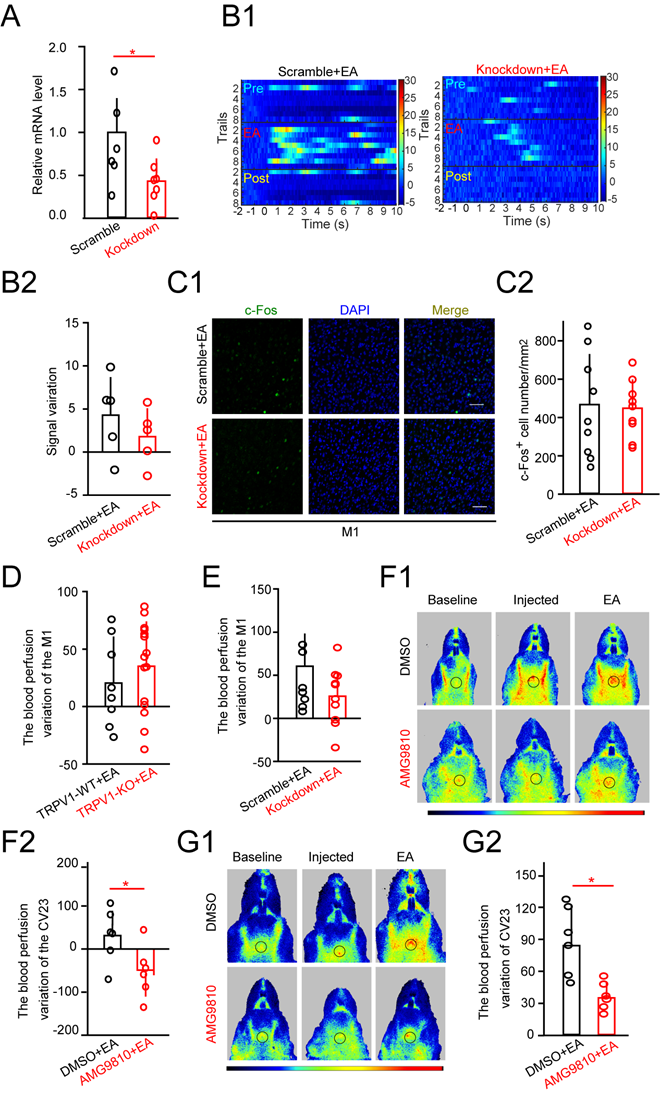


**Supplementary figure4:**

**(A)** The RT-PCR revealed a decreased expression of TRPV1 in TRPV1-Knockdown mice compared to that in the TRPV1-Scramble mice. Two-tailed Student’s unpaired t-test, n=7 per group, **P* <0.05.

**(B)** Representative heatmap of CaMKIIα^+^ neurons in the M1 of Scramble and Knockdown virus injection locally at the CV23. The results showed that the relative change of fluorescence in the M1 during Pre, EA, and Post conditions from the Scramble+ EA group and Knockdown+ EA group **(B1)**. Quantification of the average amplitude of attenuated signals in the Knockdown+ EA group compared to that in the Scramble+ EA group **(B2)**. n= 6 per group.

**(C)** Representative images of c-Fos in the M1 following EA treatment after intramuscular injection of Scramble or Knockdown virus at the CV23 **(C1)**. Scale bars, 100 µm. A bar graph showed that the density of c-Fos in the M1 following EA treatment wasn’t changed in the Knockdown+ EA group compared to that in the Scramble+ EA group **(C2)**. n=9 per group.

**(D)** A bar graph revealed no change in blood perfusion variation of the M1 after EA treatment in the TRPV1-KO group compared to that in the TRPV1-WT group. n= 8 ~ 17.

**(E)** A bar graph revealed no change in blood perfusion variation of M1 after EA treatment in the TRPV1-Kockdown group compared to that in the TRPV1-Scramble group. n = 6 ~ 10.

**(F)** Representative LSCI showed the blood perfusion of the lower jaw in baseline, injected, and EA conditions from DMSO and AMG9810 groups **(F1)**. The drug was injected into the CV23 acupoint. Time: 120 s, black area: CV23, target area = 4 mm^2^. The graphical representation revealed that the hemoperfusion variability exhibited a significant reduction in the AMG9810+EA cohort relative to that observed in the DMSO+EA group **(F2)**. Two-tailed Student’s unpaired t-test, n =6 per group, **P* <0.05.

**(G)** Representative LSCI showed the blood perfusion of the lower jaw in baseline, injected, and EA conditions from DMSO and AMG9810 groups **(G1)**. The drug was administrated by intraperitoneal injection. Time: 120 s, black area: CV23, target area = 4 mm^2^. The graphical representation revealed that the hemoperfusion variability exhibited a significant reduction in the AMG9810+EA cohort relative to that observed in the DMSO+EA group **(G2)**. Two-tailed Student’s unpaired t-test, n =6 per group, **P* <0.05.

Data are shown as mean ± SD.

**Supplementary figure5**
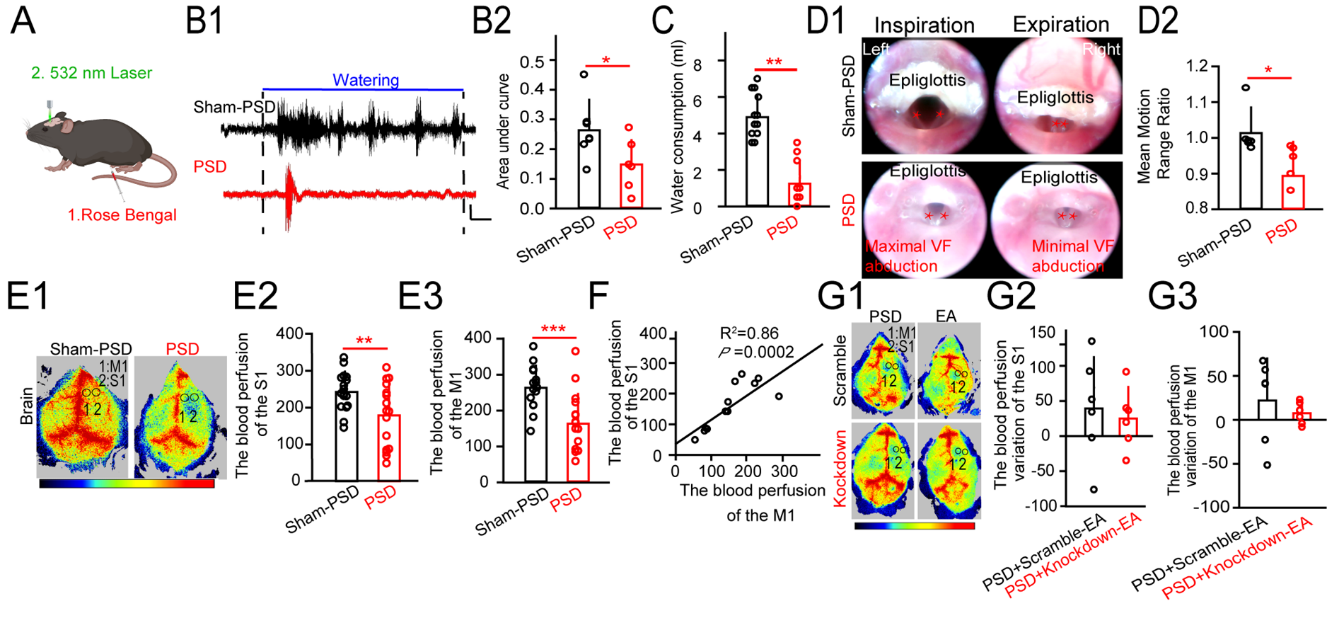


**Supplementary figure5:**

**(A)** The strategy to establish a PSD mouse model.

**(B)** Example traces of EMG response to watering stimulation in the Sham-PSD group and PSD group **(B1)**. Scale bars, time = 1 s, bin = 0.1 mv. The quantification of AUC for EMG response from the lower muscle was assessed in both Sham-PSD and PSD groups **(B2)**. The result showed that the swallowing function was impaired in the PSD group compared to that in the Sham-PSD group. Two-tailed Student’s unpaired t-test, n =6~7, **P* <0.05.

**(C)** A bar graph revealed water consumption in the different groups. The results showed that the swallowing function was impaired in the PSD group compared to that in the Sham-PSD group. Two-tailed Student’s unpaired t-test, n =14 per group, ***P* <0.01.

**(D)** Representative endoscopic images of the murine larynx, captured at maximum and minimum vocal fold (VF) abduction during spontaneous breathing from a 30-fps video, were thoroughly scrutinized. Eminent laryngeal structures of interest, including the bilateral VFs labeled with striking red asterisks, and the epiglottis, were meticulously inspected **(D1)**. Quantification showed that the mean motion range ratio (MMRR) in the PSD group was shorter than that in the Sham-PSD group **(D2)**. Two-tailed Student’s unpaired t-test, n =6 per group, **P* <0.05.

**(E)** Representative LSCI of the brain in the Sham-PSD and the PSD group **(E1)**. Time: 120 s, target 1: M1, target 2: S1, target area = 0.5 mm^2^. A bar graph showed that the blood perfusion of M1 was lower in the PSD group than that in the sham-PSD group **(E2)**. Two-tailed Student’s unpaired t-test, n =15~19, ***P* <0.01. A bar graph showed that the blood perfusion of S1 was lower in the PSD group than that in the sham-PSD group **(E3)**. Two-tailed Student’s unpaired t-test, n =15~19, ****P* <0.001.

**(F)** Positive correlation between the perfusion of M1 and S1 analyzed by laser speckle. A significantly positive statistical correlation was found between the groups. R^2^= 0.86, *P* < 0.001.

**(G)** Representative LSCI showed the blood perfusion of brains in PSD and PSD+ EA conditions from TRPV1-Scramble and TRPV1-Kockdown groups **(G1)**. Time: 120 s, target 1: M1, target 2: S1, target area = 0.5 mm^2^.A bar graph revealed no difference in blood perfusion of S1 after PSD with EA treatment in the TRPV1-Kockdown group compared to that in the TRPV1-Scramble group **(G2)**. n =6 per group. A bar graph revealed no difference in blood perfusion of M1 after PSD with EA treatment in the TRPV1-Kockdown group compared to that in the TRPV1-Scramble group **(G3)**. n =6 per group.

Data are shown as mean ± SD.

**Supplementary figure6**


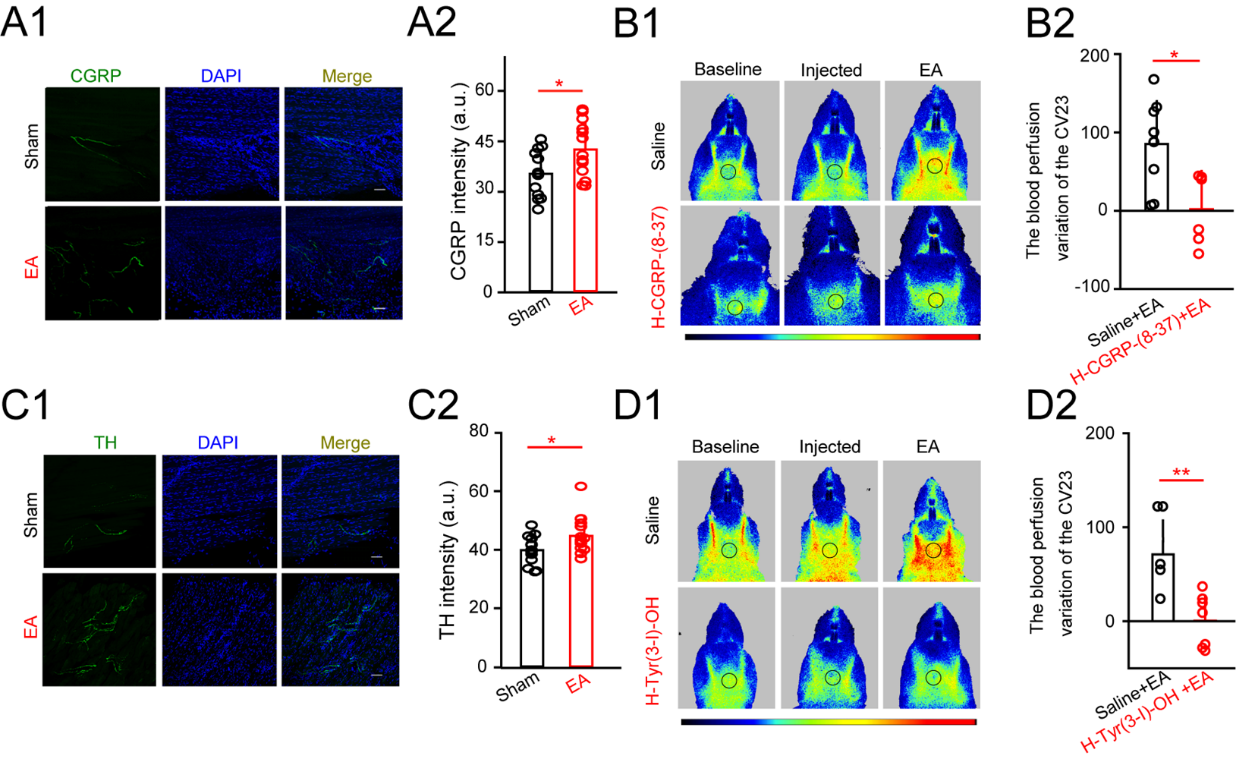


**Supplementary figure6:**

**(A)** Representative images of immunofluorescent staining of CGRP in the Sham and EA groups **(A1)**. Quantification analysis showed that the intensity of CGRP was significantly increased in the EA group compared to that in the Sham group **(A2)**. Two-tailed Student’s unpaired t-test, n (slices) =13~14, **P* <0.05.

**(B1)** Representative LSCI showed the blood perfusion of the lower jaw in baseline, injected, and EA conditions from saline and H-CGRP-(8-37) groups. The drug was injected into the CV23 acupoint. Time: 120 s, black area: CV23, target area = 4 mm^2^.

A bar graph showed that the blood variation perfusion was decreased in the Saline+ EA group compared to that in the H-CGRP-(8-37) +EA group **(B2)**. Two-tailed Student’s unpaired t-test, n =6~8, **P* <0.05.

**(C)** Representative images of immunofluorescent staining of TH in the Sham and EA groups **(C1)**. Quantification analysis showed that the intensity of TH was significantly increased in the EA group compared to that in the Sham group **(C2)**. Two-tailed Student’s unpaired t-test, n (slices) =13 per group, **P* <0.05.

**(D)** Representative LSCI showed the blood perfusion of the lower jaw in baseline, injected, and EA conditions from saline and H-Tyr(3-I)-OH groups **(D1)**. The drug was injected into the CV 23 acupoint. Time: 120 s, black area: CV23, target area = 4 mm^2^. A bar graph showed that the blood variation perfusion was decreased in the DMSO+EA group compared to that in the H-Tyr(3-I)-OH+ EA group **(D2)**. Two-tailed Student’s unpaired t-test, n =6~7, ***P* <0.01.

Data are shown as mean ± SD.
